# Sleep duration, snoring habits and risk of acute myocardial infarction in China population: results of the INTERHEART study

**DOI:** 10.1186/1471-2458-14-531

**Published:** 2014-05-29

**Authors:** Dongfang Xie, Wei Li, Yang Wang, Hongqiu Gu, Koon Teo, Lisheng Liu, Salim Yusuf

**Affiliations:** 1 Division of Biometrics, National Center for Cardiovascular Diseases, Cardiovascular Institute & Fu Wai Hospital, Peking Union Medical College & Chinese Academy of Medical Sciences, Beijing 100037, China; 2 Beijing Hypertension League Institute, Beijing, 100039, China; 3 Population Health Research Institute, McMaster University, Hamilton, Canada

**Keywords:** Cardiovascular disease, Snoring, Sleep, Acute myocardial infarction, China

## Abstract

**Background:**

Less sleep time and snoring have been associated with cardiovascular disease (CVD) risk in Western populations; however, few studies have evaluated the different aspects of sleep duration and snoring frequency in relation to CVD, and this association has not been examined in China. The present study aimed to address the relation between sleep duration, snoring frequency and risk of acute myocardial infarction (AMI) in China population.

**Methods:**

We conducted a hospital-based case–control study. Cases were first AMI (*n* = 2909). Controls were matched to cases on age and sex. 2947 controls who did not report previous angina or physical disability completed a questionnaire on sleep duration and snoring frequency. We used logistic regression to control for other risk factors.

**Results:**

We observed an inverse association between serious snoring frequency and AMI risk. After adjustment for all the risk factors, and the OR for everyday group and 3–5 times per week group was 1.45 (95% CI: 1.01 to 1.91) and 1.93 (95% CI: 1.52-2.46) compared to no snoring group. The OR for serious level group and moderate group was 1.77 (95% CI: 1.29 to 2.43) and 1.37 (95% CI: 1.10 to 1.69) compared to no snoring group. People having serious snoring increased 77% risk of AMI. 15.2% people in control group have ≤ 6 hours sleeping, compared with 17.4% in AMI group.

**Conclusions:**

Snoring frequency, including as much as everyday and 3–5 times per week, was positively associated with AMI risk and less sleep duration was associated with risk of AMI. Less sleep time could increase AMI risk in China population.

## Background

Cardiovascular disease (CVD) is the leading cause of death and disability-adjusted life-years world wide, with increasing incidence and prevalence in low- and middle-income countries. By 2020, more than 80% of global CVD will be in these countries, with the largest burden occurring in the two largest countries, China and India, as they rapidly urbanise [1].

Sleep duration has been associated with CVD, stroke, diabetes, hypertension, and all-cause mortality [2-13]. A recent meta-analysis documented that compared with 7 hours of sleep, shorter duration of sleep was associated with up to a 2-fold increased risk for CVD-related mortality [2]. For both men and women, lack of sleep has been associated with increased risk of cardiovascular events [5,6]. Amagai et al. 3 showed that sleeping less than 6 hours per night, when compared with sleeping 7 to 7.9 hours per night, was associated with CVD risk of 2.14 for men and 1.46 for women. Understanding the relation between lack of sleep and adverse health effects is important because over the last 30 years, diminished sleep duration (< 6 hours) has increased from 7.6% of the population in 1975 to 9.3% in 2006 [14]. Potential mechanisms for the relation between inadequate sleep and increased cardiovascular risk include up-regulation of appetite and decreased energy expenditure, leading to obesity and its metabolic consequences, and alterations in glucose metabolism [15,16].

Associations of snoring with cardiovascular disease was examined cross-sectionally [17,18] in clinical and large-scale epidemiologic studies, most of which clearly showed an independent positive association, even after adjustment for potential confounding factors such as age, sex, and body mass index (BMI) [19]. This causal relationship has also been observed in studies of Western populations. As compared with non-snorers, the relative risk of developing cardiovascular disease among habitual snoring American women was 30% higher in the Nurses’ Health Study [20], and the risk among habitual and frequent snoring Finish men was 2.1-fold higher [21]. However, evidence from Asian populations is still very limited, and these results from Western studies cannot simply be extrapolated to Asian populations.

The purpose of this study was to assess the association between sleep habits, defined as sleep duration and snoring, and measured risk factors for CVD in China population, including both traditional and psychosocial risk factors. An additional aim of the research was to evaluate the potential mechanisms related to sleep habits and cardiovascular risk as well as to assess any interaction by gender or age.

## Methods

### Study population

INTERHEART China study is a case–control study. In the study, 2909 patients with incident cases of acute myocardial infarction (AMI) and 2947 control subjects free of heart disease from 27 regions in China were enrolled between February 1999 and March 2003.

To identify first cases of acute myocardial infarction, all patients (irrespective of age) admitted to the coronary care unit or equivalent cardiology ward, presenting within 24 h of symptom onset, were screened. Cases were eligible if they had characteristic symptoms plus electrocardiogram changes indicative of a new myocardial infarction. At least one age-(±5 years) and sex-matched control (without a history of CVD) was recruited for each case from non-cardiac wards or unrelated visitors of cardiac patients, or patients at the same centers with illnesses not obviously related to CVD or its risk factors. At entry to the study, informed consent was obtained from each subject. This study was approved by appropriate regulatory and ethics councils in all participating centers. A structured and pre-tested questionnaire was administered and physical examinations were undertaken in the same manner among cases and controls [22-24].

### Ethical approval and informed consent

Subjects willing to participate are subsequently sent written information on the study procedures along with an informed consent form. Only participants providing written informed consent are enrolled. Ethical approval was obtained by the Medical Ethical Committee of Beijing Fu Wai Hospital.

### Procedures

Trained personnel administered the structured questionnaires and physical examinations in a standardized manner. Information about demographic factors, family income and education, lifestyle, personal and family history of CVD and risk factors, psychosocial factors, physical activities, and smoking history, alcohol use were obtained with the use of standardized INTERHEART questionnaires. Personal and family patterns of cardiovascular disease (CVD) and risk factors were recorded, and histories of hypertension and diabetes were self-reported. Three categories of educational level were created: none or 1–8 years, 9–12 years, trade school or college or university. Family income defined as three groups. Three categories were lower group (≤ 6000 yuan/year), middle group (6000–10000 yuan/year), and higher group (≥ 10000 yuan/year) according to the level of income. Questions were included about psychosocial conditions to identify depressive mood and psychological stress. General psychological stress was defined as experiencing stress at work or at home and was also assessed in the four categories. Subjects were asked whether they had suffered from some Stressful life events in the previous year, including marital separation/divorce, loss of job/retirement, loss of crop/business failure, violence, major intra-family conflict, major personal injury or illness, death/major illness of a close family member, death of a spouse and other major stress. Blood pressure, height, weight, waist and hip circumferences were measured. Body mass index (BMI) was defined as weight (kg) divided by squared height (m^2^).

During the INTERHEART examination, a self-administered sleep history questionnaire was obtained. Among the questions were a) “Have you ever snored (now or at any time in the past)?”; if yes, “How frequent and loudly do you snore?”; b) “How long your night’s sleep?” Participants were provided 4 responses to each question: ≤ 6, 6–8, > 8, don’t know; and c) “Do you have the habit of napping?”; if yes, “How long do you nap everyday?”. Among the INTERHEART participants, who did not report “don’t know” to either the sleep or snoring questions and were therefore excluded from this analysis.

### Statistical analysis

Clinical data of continuous variables expressed as mean (SD) and differences were assessed by rank test. Categorical variables were represented as percentage and were tested by Pearson chi-squared analysis.

The association between sleep, snoring frequency and AMI was assessed in several different ways. In models which adjusted for age, sex and area, we investigated the effect on each variable on the risk of AMI. Subsequently, multivariable logistic regression models were built where, after adjustment for age and sex, we separately introduced (1) age, sex, BMI, hypertension, diabetes, stroke and family history of MI. (2) age, sex, area, WHR, BMI, smoking, drinking, physical activity, consumption of vegetables and fruits. (3) age, sex, area, educational status, household income, marital status, stress and depression. Age, BMI, and whr levels were approximate a normal distribution. *P* < 0.05 was used to indicate statistically significant differences. Significance was assessed by a Wald test and by a likelihood-ratio test.

During this process, we explored the interaction between snoring frequency and other risk factors. The Wald chi-square test was used to test the interaction.

All statistical tests were two-sided. Statistical analyses and graphics were produced with the SAS software version 9.1.3 and Excel.

## Results

In the INTERHEART China study, cases and controls, matched by age and gender, were enrolled between February 1999 and March 2003 from 27 regions in China. Characteristics of Participants were shown in Table 1. History of hypertension, diabetes and stroke, smoking, education status, income, general stress, dietary frequency and both higher WHR and BMI levels were significant determinants of AMI in this population.

**Table 1:** Characteristics of participants

**Characteristics**	**AMI cases,** ***N*** **= 2909**	**Controls,** ***N*** **= 2947**	***χ***^**2**^**/*Z***	***P*-value**
Male sex (%)	2027 (69.7)	2048 (69.5)	0.02	0.8771
Mean Age (SD), year	62.1 (11.7)	60.3 (11.4)	6.28	0.0001
BMI, n (%)			18.40	0.0001
≤ 24	1173 (40.3)	1341 (45.5)		
24-28	1358 (46.7)	1291 (43.8)		
> 28	378 (13.0)	315 (10.7)		
WHR, mean (SD), cm/cm	0.88 (0.08)	0.88 (0.08)	2.16	0.2011
Hypertension (%)	1145 (39.5)	656 (22.26)	203.68	0.0001
Diabetes (%)	360 (12.4)	87 (3.0)	185.54	0.0001
Stroke (%)	317 (10.9)	102 (3.5)	122.76	0.0001
Family history of MI, n (%)	102 (3.5)	54 (1.8)	15.93	0.0001
Smoking, n (%)	1694 (58.2)	1186 (40.2)	189.54	0.0001
Alcohol, n (%)	463 (15.9)	429 (14.6)	2.09	0.1479
Physical activity, n (%)	128 (14.8)	558 (19.0)	17.93	0.0001
Educational status, n (%)			52.57	0.0001
Trade school/college/university	553 (19.1)	723 (24.5)		
9-12 years	799 (27.5)	924 (31.4)		
≤ 8 years	1546 (53.4)	1300 (44.1)		
Income			10.72	0.0047
Higher	1021 (35.3)	1157 (39.2)		
Middle	928 (32.0)	907 (30.8)		
Lower	941 (32.7)	875 (30.0)		
Single or divorced, n (%)	366 (12.6)	197 (6.7)	58.81	0.0001
General stress, n (%)			20.33	0.0001
Permanent	1083 (37.4)	944 (32.0)		
Several periods	1311 (45.3)	1411 (47.9)		
Never experienced	500 (17.3)	591 (20.1)		
Depressed, n (%)	559 (19.6)	273 (9.3)	122.88	0.0001
Consumption of vegetables (times/week)	7.8 (5.5)	7.9 (5.4)	−1.10	0.2705
Consumption of fruits (times/week)	4.0 (3.7)	4.8 (3.7)	−9.29	0.0001

*N*, number; *p*-value, significance of difference between group means and frequency determined by rank-test and chi-square test.

Age-, sex- and area-adjusted and multivariable adjusted Odds ratio (OR) for AMI risk factors are presented in Table 2. After adjustment for age and sex, the OR associated with nap after lunch, compared with no nap was 1.06 (95% CI: 0.96 to 1.18). The model adjusted for all variables, the ORs were 0.94 (95% CI: 0.84 to 1.07).

**Table 2:** Odds Ratios (95% confidence intervals) for Acute Myocardial Infarction (AMI) risk for sleep and snoring with different levels of adjustments

**Sleep and snoring**	**Case (%)**	**Control (%)**	Odds ratio (95% CI)*	**Adjusted OR (95% CI)**^**†**^	**Adjusted OR (95% CI)**^**‡**^	**Adjusted OR (95% CI)**^**§**^	**Adjusted OR (95% CI)**^**||**^
**Nap after lunch**							
No	1384 (47.8)	1488 (50.5)	1.00	1.00	1.00	1.00	1.00
Yes	1510 (52.2)	1458 (49.5)	1.06 (0.96-1.18)	1.00 (0.89-1.11)	1.05 (0.94-1.17)	1.00 (0.89-1.11)	0.94 (0.84-1.07)
**Snoring frequency**							
No snoring	546 (19.2)	725 (25.0)	1.00	1.00	1.00	1.00	1.00
0-1 time per week	635 (22.4)	796 (27.4)	1.06 (0.91-1.24)	1.08 (0.92-1.26)	0.94 (0.80-1.10)	1.06 (0.90-1.24)	0.94 (0.80-1.12)
1-3 times per week	1115 (39.3)	1069 (36.8)	1.40 (1.21-1.62)	1.33 (1.15-1.55)	1.29 (1.11-1.50)	1.37 (1.18-1.60)	1.20 (1.02-1.42)
> 3 times per week	542 (19.1)	313 (10.8)	2.31 (1.93-2.76)	1.92 (1.59-2.31)	2.03 (1.67-2.46)	2.17 (1.80-2.62)	1.72 (1.40-2.11)
**Snoring severity**							
No snoring	532 (19.4)	732 (26.1)	1.00	1.00	1.00	1.00	1.00
Mild	1647 (59.9)	1685 (60.1)	1.35 (1.18-1.54)	1.32 (1.14-1.51)	1.22 (1.06-1.41)	1.33 (1.16-1.53)	1.16 (1.00-1.35)
Moderate	404 (14.7)	298 (10.6)	1.87 (1.55-2.26)	1.60 (1.32-1.95)	1.66 (1.36-2.03)	1.77 (1.45-2.15)	1.37 (1.10-1.69)
Serious	166 (6.0)	87 (3.1)	2.70 (2.03-3.59)	2.08 (1.55-2.80)	2.18 (1.62-2.95)	2.65 (1.98-3.55)	1.77 (1.29-2.43)

*Model adjusted for age, sex and area.

^†^Model adjusted for age, sex, area, hypertension, diabetes, stroke and family history of MI.

^‡^Model adjusted for age, sex, area, whr, bmi, smoking, drinking, physical activity, consumption of vegetables and fruits.

^§^Model adjusted for age, sex, area, educational status, household income, marital status, stress and depression.

^||^Model adjusted for all the risk factors.

After adjustment for age and sex, the OR associated with snoring everyday, compared with no snoring was 2.06 (95% CI: 1.61 to 2.63), for 3–5 times per week group was 2.50 (95% CI: 2.02 to 3.11), for 1–3 times per week group was 1.40 (95% CI: 1.21 to 1.62), and for 0–1 time per week group was 1.06 (95% CI: 0.91 to 1.24). After adjustment for age, sex, area, whr, bmi, smoking, drinking, physical activity, consumption of vegetables and fruits, these estimates were slightly decreased (OR: 1.80, 95% CI: 1.39 to 2.33 for everyday group; OR: 2.20, 95% CI: 1.75 to 2.77 for 3–5 times per week group; OR: 1.29, 95% CI: 1.11 to 1.50 for 3–5 1–3 times per week; OR: 0.94, 95% CI: 0.80 to 1.10 for 0–1 time per week group). In the final model, adjusted for all the risk factors, and the OR for everyday group and 3–5 times per week group was 1.45 (95% CI: 1.01 to 1.91) and 1.93 (95% CI: 1.52-2.46) compared to no snoring group.

In age-, sex- and area-adjusted analysis of snoring severity (Table 2), people in the highest level (serious snoring) had an OR of 2.70 (95% CI: 2.03 to 3.59; *P* value = 0.0001) compared with those in the lowest level (no snoring). The association remained similar in multivariate analysis. After adjustment for age, sex, area, educational status, household income, marital status, stress and depression, OR associated with serious level group was 2.65, compared with no snoring group. After adjustment for age, sex, area, whr, bmi, smoking, drinking, physical activity, consumption of vegetables and fruits, with serious level group having an 20% decreased risk compared with the no snoring group (OR = 2.18; 95% CI: 1.62 to 2.95; *P* value = 0.0001). In final analysis adjusted for all the risk factors, the OR for serious level group and moderate group was 1.77 (95% CI: 1.29 to 2.43) and 1.37 (95% CI: 1.10 to 1.69) compared to no snoring group. People having serious snoring increased 77% risk of AMI.

The association of time of sleep and AMI are presented in Table 3. 67.4% people in control group have 6–8 hours sleeping, compared with 62.2% in AMI group (*P*-value, 0.0002). 15.2% people in control group have ≤ 6 hours sleeping, compared with 17.4% in AMI group.

**Table 3:** Sleep duration difference between Acute Myocardial Infarction (AMI) participants and controls

**Nap and sleep duration**	**AMI cases**	**Controls**	***χ***^**2**^	***P*-value**
Nap duration (minutes)	*N* = 1509	*N* = 1455	25.42	0.0001
≤ 30	384 (25.5)	489 (33.6)		
30-60	791 (52.4)	704 (48.4)		
> 60	334 (22.1)	262 (18.0)		
Sleep duration (hours)	*N =* 2873	*N* = 2918	17.09	0.0002
≤ 6	499 (17.4)	444 (15.2)		
6-8	1787 (62.2)	1966 (67.4)		
> 8	587 (20.4)	508 (17.4)		

*N*, number; *p*-value, significance of difference between group frequency determined by chi-square test.

Age-, sex- and area-adjusted and multivariable adjusted Odds ratio (OR) for snoring associated factors were presented in Table 4. After adjustment of all other variables, BMI, hypertension, smoking, and general stress were associated with AMI.

**Table 4:** Associations between Snoring and Acute Myocardial Infarction (AMI) risk factors

**AMI related factors**	**Snoring (Y/N)**		
	***P*** value^*^	Odds ratio (95% CI)*	**Adjusted OR (95% CI)**^**†**^
Sex (Male vs Female)	0.0002	1.40 (1.18-1.68)	1.43 (1.14-1.79)
BMI	0.0001		
≤24		1.00	1.00
24-28		1.79 (1.49-2.14)	1.80 (1.48-2.18)
>28		2.53 (1.82-3.51)	2.51 (1.75-3.58)
Hypertension (Yes vs No)	0.0001	1.84 (1.47-2.30)	1.52 (1.17-1.97)
Diabetes (Yes vs No)	0.9045	1.03 (0.63-1.70)	0.88 (0.50-1.52)
Stroke (Yes vs No)	0.0332	1.80 (1.05-3.09)	0.83 (0.46-1.50)
family history of MI (Yes vs No)	0.8834	1.05 (0.56-1.97)	1.13 (0.57-2.24)
Smoking (Yes vs No)	0.0001	1.89 (1.58-2.27)	1.88 (1.51-2.34)
Alcohol (Yes vs No)	0.0002	1.66 (1.27-2.17)	1.30 (0.97-1.76)
Physical activity (Yes vs No)	0.6928	0.96 (0.77-1.19)	1.10 (0.86-1.39)
Single or divorced (Single vs married)	0.0004	0.58 (0.42-0.78)	0.57 (0.40-0.81)
General stress	0.0001		
Permanent		1.00	1.00
Several periods		2.13 (1.73-2.63)	1.89 (1.49-2.38)
Never experienced		2.31 (1.83-2.90)	2.16 (1.67-2.79)
Depressed (Yes vs No)	0.2133	0.83 (0.63-1.11)	0.92 (0.68-1.26)

^*^Calculated by Chi-squire test.

*Univariate association analysis by logistic regression.

^†^Model adjusted for all the risk factors.

Table 5 showed the interaction between snoring frequency and related factors of AMI in the INTERHEART China study. The interaction between levels of snoring frequency and permanent stress was significant (*P* value for interaction, 0.0155).

**Table 5:** Interaction between snoring frequency and other related factors of AMI

	**No snoring**	**0-1 time per week**	**1-3 times per week**	**>3 times**	***P*-value for interaction**
Age (years), mean (SD)					0.0133
Case	62.5 (12.4)	61.8 (11.1)	62.5 (11.7)	60.9 (11.4)	
Control	59.5 (12.7)	59.9 (10.5)	61.0 (11.4)	60.5 (11.0)	
BMI (kg/m2), mean (SD)					0.7755
Case	24.0 (3.4)	24.5 (3.4)	24.8 (3.1)	25.6 (3.3)	
Control	23.6 (3.0)	24.7 (2.7)	24.6 (2.8)	25.3 (3.9)	
WHR, mean (SD)					0.0179
Case	0.87 (0.08)	0.89 (0.07)	0.86 (0.09)	0.91 (0.08)	
Control	0.88 (0.08)	0.89 (0.08)	0.86 (0.09)	0.90 (0.07)	
Hypertension, *n* (%)					0.2176
Case	190 (34.8)	198 (31.2)	450 (40.4)	289 (53.3)	
Control	111 (15.3)	153 (19.2)	286 (26.8)	105 (33.6)	
Diabetes, *n* (%)					0.8630
Case	58 (10.6)	63 (9.9)	153 (13.7)	76 (14.0)	
Control	21 (2.9)	18 (2.3)	35 (3.3)	12 (3.8)	
Stroke, *n* (%)					0.2193
Case	42 (7.7)	54 (8.5)	155 (13.9)	63 (11.7)	
Control	16 (2.2)	19 (2.4)	47 (4.4)	19 (6.1)	
Current smokers, *n* (%)					0.1616
Case	242 (44.3)	356 (56.1)	655 (58.7)	404 (74.5)	
Control	214 (29.5)	325 (40.8)	462 (43.2)	176 (56.2)	
Alcohol consumption (≥1/week), *n* (%)					0.8611
Case	50 (9.2)	85 (13.4)	198 (17.8)	121 (22.3)	
Control	74 (10.2)	82 (10.3)	186 (17.4)	78 (24.9)	
Physical activity, *n* (%)					0.7550
Case	93 (17.0)	65 (10.3)	122 (11.0)	132 (24.4)	
Control	137 (18.9)	117 (14.7)	196 (18.3)	84 (27.0)	
Education (≤8 years), *n* (%)					0.5209
Case	292 (53.5)	340 (53.6)	631 (56.6)	253 (46.7)	
Control	308 (42.5)	343 (43.3)	520 (48.6)	114 (36.4)	
Low family income, *n* (%)					0.8004
Case	188 (34.6)	200 (31.6)	323 (31.7)	175 (32.4)	
Control	221 (30.7)	242 (30.4)	321 (30.1)	76 (24.3)	
Permanent stress, *n* (%)					0.0148
Case	199 (36.5)	262 (41.3)	399 (35.8)	193 (35.7)	
Control	194 (26.8)	263 (33.0)	369 (34.6)	101 (32.3)	
Depression, *n* (%)					0.5744
Case	113 (21.3)	118 (18.9)	179 (16.2)	127 (23.8)	
Control	75 (10.5)	70 (8.9)	77 (7.3)	45 (14.4)	
Vegetables consumption, (times/week), mean (SD)					0.0001
Case	7.1 (5.3)	6.6 (5.0)	8.2 (5.5)	9.3 (6.0)	
Control	7.7 (5.5)	7.5 (4.9)	8.3 (5.4)	8.4 (5.8)	
Fruit consumption, (times/week), mean (SD)					0.0270
Case	3.5 (3.5)	3.5 (3.2)	4.5 (3.5)	4.2 (4.4)	
Control	4.4 (4.0)	4.5 (3.3)	5.3 (3.5)	4.6 (3.8)	

**Figure 1:**
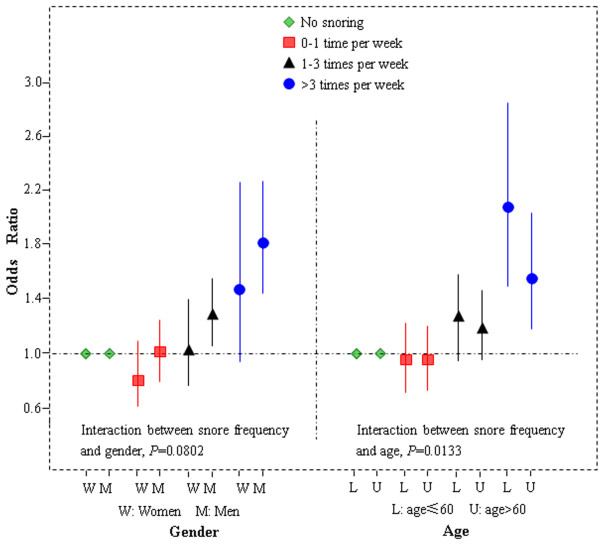
Adjusted odds ratios (and 95% confidence intervals), with tests for heterogeneity for risk of acute myocardial infarction for snore frequency by different type of gender and age.

Figure 1 showed the association of snoring frequency with risk for AMI in men versus women. In models adjusted for all the risk factors, OR associated with snoring frequency (3–5 times per week group, compared to no snoring group), was 2.26 (95% CI: 1.30 to 3.92) in women, 1.90 (95% CI: 1.44 to 2.50) in men, (*p* value for heterogeneity, 0.1059). The effect of snoring was stronger in younger population; the OR associated with snoring frequency (everyday group, compared to no snoring group), was 1.88 (95% CI: 1.25 to 2.83) in younger, 1.18 (95% CI: 0.81 to 1.73) in older. *p*-value was significant; *p* = 0.0201.

## Discussion

In the present analysis, we evaluated the association between categories of sleep duration and snoring frequency as well as the duration of activity with AMI risk factors and to the risk of developing an AMI.

We observed an inverse association between serious snoring frequency and AMI risk after controlling for similar demographic and potential confounders. People having serious snoring increased 77% risk of AMI. Our results found that there was an increased risk for CVD in China population who slept less than 6 hours or more than 8 hours per day when compared with those who sleep 6 to 8 hours per day. Another study found that the odds of sleeping less than 6 hours per night was lower for women and married persons [14]. The Nurse’s Health Study found a modest relationship between coronary events and sleeping 6 hours or less compared with 8 hours (OR, 1.18; 95% CI, 0.98-1.42) [5]. In a Japanese population, less than 6 hours of sleep was associated with a significant 2-fold increase in CVD events in men and a non significant trend in women [4]. Our data are consistent with those of the National Health Interview Survey that showed that sleep duration was a significant predictor of CVD independent of age, gender, and race/ethnicity [2]. Lack of sleep has been linked to several CVD traditional and psychosocial risk factors, as observed in our study. Previous research has shown that sleep deprivation results in decreased glucose tolerance and elevated blood pressure [2].

In the present prospective study, snoring frequency was associated with an increased incidence of cardiovascular events among community-dwelling middle-aged Japanese women. This association was independent of age and other confounding factors. As compared with never snorers, ‘everyday snoring’ women had a 2.5-fold higher risk of cardiovascular events during 6 years of follow-up. The association of everyday snoring with cardiovascular events was attenuated after adjustment for BMI and after further adjustment for systolic blood pressure, antihypertensive medication use, diabetes mellitus, and hypercholesterolemia. This suggested that overweight partly mediated the association and that hypertension and metabolic abnormalities partly caused by snoring contribute to the risk of cardiovascular events in women who snore every day. This is the first study to show a relationship between habitual snoring and risk of cardiovascular events among a population in Asia, which has a low prevalence of obesity. The biological mechanisms that link habitual snoring to the development of cardiovascular disease remain to be fully elucidated, but a number of mechanisms have been proposed. Habitual snoring is often accompanied by sleep apnea or hypopnea. Repetitive episodes of intermittent complete and partial airway collapse during sleep result in hypoxemia, hypercapnia, changes in intrathoracic pressure, and repeated arousal from sleep. Episodes of snoring and apneic events can cause acute hemodynamic changes (such as increased cardiac output, enhanced cardiac arrhythmia, patent foramen ovale appearance, increased intracranial pressure, and decreased cerebral blood flow) [25], increased platelet aggregation and fibrinogen concentrations [26,27], and decreased fibrinolysis, which directly affect the cardiovascular system. Abnormal metabolic conditions such as hypertension, diabetes mellitus, and hypercholesterolemia may also increase the risk of cardiovascular disease via elevation of sympathetic activation, oxidative stress [28-30], activation of the hypothalamic-pituitary-adrenal axis due to sleep fragmentation, and endothelial dysfunction [31-33].

In our study, we observed snoring frequency was more significant associated with AMI in men, compared to women in China population. In contrast to the significant association of habitual snoring with cardiovascular events in women, no such association was observed in men. Large population-based prospective studies of middle-aged men, women, and a population of men and women aged 20 years or older have reported positive associations between habitual snoring and cardiovascular events [21,22]. However, no study has reported a sex difference in the association. Recent reports from the Sleep Heart Health Study (a large population-based study of American residents aged 40 or older) have noted sex differences in the association between SDB, as defined by the apnea-hypopnea index (AHI), and the risk of coronary heart disease, heart failure, and stroke [34,35]. The multivariable HRs associated with a 10-unit increase in AHI were 1.1 (1.0-1.3) for incident heart failure in men and 1.1 (1.0-1.2) for incident coronary heart disease in men aged 70 years or younger, whereas no such associations were observed in women [21]. Similarly, the multivariable HR for ischemic stroke incidence was 2.9 (1.1-7.4) in men and 1.2 (0.7-2.2) in women for the highest (>19) as compared with the lowest (≤4) AHI quartiles [22]. The reasons for the present lack of association between habitual snoring and risk of cardiovascular events in men are unknown. We found stronger association in men than women when the analysis was stratified by age, smoking, or drinking status. Further research is necessary to elucidate this sex difference.

Several limitations of this study should be considered. First, a large potential disadvantage was that this control population may not be representative of the general Chinese population, nor were the AMI cases representative of all Chinese AMI patients. And all the individuals were drawn from urban communities, so the study results may not necessarily apply to rural regions. Other potential sources of bias in our investigation include the selection of controls and a differential recall among cases compared with control subjects. While the use of population based controls would be ideal, we addressed selection bias that may arise with use of hospital-based controls by obtaining control subjects from other different outpatient clinics and inpatient wards in the hospital. Therefore, if an association exists between the exposure of interest and the disease status of one control group, the bias that may result would be diluted. Additionally, we used a systematic method of control selection that would avoid arbitrary selection of controls within wards and clinics. Differential recall of sleep duration and snoring frequency is also a potential concern; however, research assistants asked case subjects to specifically report on their level of sleep duration and snoring frequency prior to their myocardial infarction. While the potential for recall bias may exist, awareness of heart disease prevention and health consciousness may not be as high among our study population in China as its is in Western countries; hence the likelihood of recall bias may be lower. We also excluded all those with any prior heart disease from the study. As well, while we cannot exclude the possibility that not all sleep and snoring condition was reported, interviewers were trained to conduct thorough interviews on sleep and snoring during the day or night. While future prospective research within China will adequately address these biases, the case–control design had the advantage of being cost, resource, and time efficient.

In the further research, we need to determine the mechanisms of this association exactly. New study designs and methods need to be developed. Testing the theoretical models of potential mechanisms is highly dependent on the quality of study design and the data that results. One serious problem with the existing studies is retrospective. New epidemiological methods have been developed to overcome or evaluate some existing problems. The prospective research about the impact of physical activities on health outcomes at different economic development levels is currently being studied in the Prospective Urban Rural Epidemiologic study (PURE study) [36-38]. Further, there may be heterogeneity between physical activities and health outcomes across different populations through 20 years follow-up. In PURE study, more fine measures of physical activities during the life course are needed.

## Conclusions

Snoring frequency, including as much as everyday and 3–5 times per week, was positively associated with AMI risk and less sleep duration was associated with risk of AMI. Less sleep time could increase AMI risk in China population.

## Competing interests

The INTERHEART study was funded through unrestricted grants from several pharmaceutical companies (with major contributions from AstraZeneca, Novartis, Hoechst Marion Roussel (now Aventis), Knoll Pharmaceuticals (now Abbott), Bristol Myers Squibb, King Pharma and Sanofi-Sythelabo).

## Authors’ contributions

DX carried out the analysis of association between snoring and AMI studies, participated in INTERHEART China study. YW and HG performed the statistical analysis. SY and KT participated in the design of the study. WL and LL conceived of the study, and participated in its design and coordination and helped to draft the manuscript. All authors read and approved the final manuscript.

## Pre-publication history

The pre-publication history for this paper can be accessed here:

http://www.biomedcentral.com/1471-2458/14/531/prepub
